# Hydroxytyrosol Ameliorates Colon Inflammation: Mechanistic Insights into Anti-Inflammatory Effects, Inhibition of the TLR4/NF-κB Signaling Pathway, Gut Microbiota Modulation, and Liver Protection

**DOI:** 10.3390/foods14071270

**Published:** 2025-04-04

**Authors:** Jiali Tang, Mengyao Zhang, Jiaying Wang, Haijing Zhang, Zhong Wang, Ziteng Lei, Chengtao Wang, Wei Chen

**Affiliations:** Key Laboratory of Geriatric Nutrition and Health, Ministry of Education, Beijing Advanced Innovation Center for Food Nutrition and Human Health, Beijing Engineering and Technology Research Center of Food Additives, School of Food and Health, Beijing Technology and Business University, No. 33 Fucheng Road, Beijing 100048, China; 2230202138@st.btbu.edu.cn (J.T.); 2230201051@st.btbu.edu.cn (M.Z.); 2230202145@st.btbu.edu.cn (J.W.); haijingvv@yeah.net (H.Z.); 2431031063@st.btbu.edu.cn (Z.W.); 2431032108@st.btbu.edu.cn (Z.L.)

**Keywords:** hydroxytyrosol, colitis, gut microbiota, liver injury, antioxidant activity, anti-inflammatory effect

## Abstract

Inflammatory bowel disease (IBD) is a chronic disease influenced by a complex interplay of factors, including genetics, environmental, and gut microbiota. This study aimed to explore the therapeutic potential of the natural polyphenolic compound hydroxytyrosol (HT) in modulating dextran sodium sulfate (DSS)-induced colitis in mice. The findings demonstrate that oral administration of HT significantly alleviated colitis symptoms, as evidenced by a reduction in the disease activity index and improvements in colonic pathology. HT was found to inhibit the release of pro-inflammatory cytokines, enhance antioxidant status, and mitigate oxidative stress. Furthermore, HT contributed to the restoration of the gut barrier by reinstating tight junction proteins, reducing the inflammatory marker lipopolysaccharide (LPS), and suppressing inflammation-related genes. This compound also modulated the NLRP3-Cas-1-GSDMD-IL-1β inflammatory pathway and inhibited the NF-κB (nuclear factor kappa B) pathway, thereby alleviating colitis. Gut microbial analysis revealed that HT enriched the abundance of Bacteroidota and altered the balance between Bacteroidota and Firmicutes in mice. Correlation analysis between bacterial microbiota and inflammatory factors suggested that HT may alleviate colitis by modulating the relative abundance of *Alistipes*, *Bacteroides*, and unclassified_f__*Muribaculaceae*. These findings underscore the potential of HT as a therapeutic agent in the treatment of colitis.

## 1. Introduction

Inflammatory bowel disease is a chronic and recurrent inflammatory condition that affects the mucosa of the gastrointestinal tract, encompassing both ulcerative colitis (UC) and Crohn’s disease (CD) [1,2]. It is one of the most prevalent and challenging diseases in gastroenterology, with its incidence rising globally. The primary clinical symptoms of IBD include diarrhea, bloody stools, and abnormal weight loss. Although the exact etiology of IBD remains unclear, it is believed to result from a combination of genetic, environmental, infectious, and immunological factors, along with dysbiosis of the gut microbiota [3,4]. A significant aspect of IBD pathogenesis involves the ecological dysregulation of critical gut microorganisms and the dysfunction of the gut barrier.

Current treatment for IBD primarily involves immunosuppressive agents, including aminosalicylates, corticosteroids, and monoclonal antibodies (e.g., infliximab). However, these treatments often come with adverse effects, including nausea, diarrhea, and abdominal pain, and they have limitations in efficacy, with some patients eventually requiring surgical intervention. Consequently, there is an urgent need to develop more effective therapeutic agents for IBD. Recently, natural bioactive substances derived from plants, such as polysaccharides [5], flavonoids [6], and plant polyphenols [7], have shown promise as potential agents for the prevention and treatment of IBD.

Hydroxytyrosol, a naturally occurring phenolic compound abundant in olive oil and olive-derived products, is characterized by a molecular formula of C8H10O_3_ and a molecular weight of 154.16 g/mol. Its chemical structure features a catechol moiety connected to an ethanol group, which confers both hydrophilic and lipophilic properties, enabling solubility in water (≥50 mg/mL) and organic solvents like ethanol. This unique amphiphilic nature contributes to its bioavailability and cellular membrane permeability. The compound demonstrates remarkable stability under acidic conditions but is susceptible to oxidation at neutral-to-basic pH, a property mitigated by its inherent antioxidant capacity through electron donation and free radical scavenging mechanisms. Hydroxytyrosol exhibits strong antioxidant activity [8]. It can be obtained through botanical extraction, chemical synthesis, and biosynthesis. Fernandez-Bolanos et al. investigated the hydrothermal treatment of two-phase olive mill waste (alperujo) and its impact on HT solubility. They successfully extracted and purified HT from alperujo using a cost-effective chromatographic system [9]. Furthermore, HT derivatives can be obtained through structural modifications such as chemical functionalization, thereby optimizing their bioactivity, stability, and targeting specificity [10]. Hydroxytyrosol and its derivatives possess a range of significant pharmacological activities, including anti-inflammatory, anticancer, antibacterial, and antiviral effects [11,12,13,14]. Consequently, HT is regarded as an excellent nutraceutical and food additive. However, its potential as a therapeutic agent for alleviating colitis has not been fully explored.

The gut microbiota is recognized as a crucial factor influencing nutrient metabolism, immune responses, and overall health [15]. It plays a significant role in maintaining the gut mucosal barrier and host immunity. The gut microbiota, predominantly composed of bacteria, also includes fungi, viruses, and helminths. The major phyla include Firmicutes, Bacteroidetes, Actinobacteria, Verrucomicrobia, and Proteobacteria [16]. In patients with inflammatory bowel disease, the composition of the gut microbiota is altered, characterized by an increase in Firmicutes and Actinobacteria, a decrease in Bacteroidetes, and changes in other phyla. Disruption of the gut microbiota and impairment of the gut barrier can lead to increased permeability and translocation of microbial products, which can then reach the liver and potentially activate the hepatic toll-like receptor 4 (TLR4) signaling pathway [17,18]. Thus, modulating the gut microbiota composition may represent an effective strategy for preventing or alleviating IBD.

Research has established the therapeutic benefits of HT in mitigating DSS-induced colitis in experimental models [19,20,21,22,23,24,25]. Nevertheless, the specific pathways responsible for these beneficial outcomes are not yet fully understood. Furthermore, the extent to which HT modulates intestinal microbial communities, the mucosal epithelial barrier, and associated metabolic profiles within murine colitis models remains incompletely characterized. Therefore, this study aims to evaluate the therapeutic effects of HT on DSS-induced intestinal injury in mice, including analyzing oxidative stress markers, monitoring blood inflammatory cytokines, investigating inflammatory pathways, assessing hepatic pyroptosis levels, exploring HT’s therapeutic pathways at the protein level, and evaluating changes in the gut microbiota. The findings will provide a theoretical foundation for the application of HT in functional foods and pharmaceutical development while elucidating its potential mechanisms of action.

## 2. Materials and Methods

### 2.1. Chemicals and Reagents

Hydroxytyrosol (98% purity), paraformaldehyde (DPEC), and phosphate-buffered saline (PBS) were procured from Beijing Half Summer Science and Technology Development Co., Ltd. (Beijing, China) and Shanghai Aladdin Biochemistry Science and Technology Co., Ltd. (Shanghai, China). Dextran sulfate sodium, with a molecular weight range of 36,000–50,000 Da, was supplied by Next Sage Biotechnology Co., Ltd. (Shanghai, China). Enzyme-linked immunosorbent assay (ELISA) kits for murine caspase-1 (Cas-1) were sourced from Jiangsu Enzyme Immunity Industry Co., Ltd. (Taizhou, China). Assay kits quantifying glutathione (GSH), malondialdehyde (MDA), superoxide dismutase (SOD), nitric oxide (NO), catalase (CAT), and total antioxidant capacity (T-AOC) were obtained from Nanjing Jiancheng Institute of Bioengineering (Nanjing, China). Additional reagents, such as lipopolysaccharide (LPS), interleukins (IL-1β, IL-6, IL-10), tumor necrosis factor-alpha (TNF-α), gasdermin D (GSDMD), glutathione peroxidase (GPX), myeloperoxidase (MPO), and hepatic biomarkers (ALT, AST, ALP, GGT), were acquired from Wuhan Fearn Biotech (Wuhan, China). All chemicals adhered to analytical-grade specifications.

### 2.2. Animals and Experimental Treatments

Specific Pathogen Free (SPF) BALB/C mice, aged 6 to 8 weeks and weighing approximately 38 ± 2 g, were sourced from the Vital River Laboratory Animal Center in Beijing, China [SCXK(Jing)2021-0006]. Following a period of acclimatization, the mice were maintained in a controlled environment with a temperature of 22 ± 2 °C, humidity levels ranging from 40% to 60%, and a light/dark cycle of 12 h.

In the experiment, 24 mice were randomly assigned to one of four groups, each consisting of six mice, namely, PBS, DSS, fecal microbiota transplantation (FMT), and HT. The PBS group was provided with unrestricted access to PBS water for the entire duration of 14 days. The DSS group received PBS water containing 3% DSS for the initial 7 days, followed by PBS water for the subsequent 7 days. In the FMT group, mice had access to 3% DSS-containing PBS water for the first 7 days, after which they were administered a daily gavage of 100 μL of FMT for the next 7 days, while having PBS water available ad libitum. The FMT was prepared by collecting fresh feces from the PBS group, homogenizing it, diluting with sterile saline to achieve a final concentration of 50 mg feces/mL, freeze-centrifuging at 4000 rpm for 2 min, and filtering through a 70 μm filter. The HT group was given PBS water with 3% DSS for the first 7 days, followed by daily gavage with HT at a dose of 60 mg/kg for the next 7 days, as established by reference to be safe without causing histopathological changes [26]. Throughout the experiment, body weights and fecal status of the mice were monitored and recorded daily.

Mice were euthanized via cervical dislocation, and blood was collected from the orbital sinus, centrifuged at 4000 rpm for 10 min, and stored at −80 °C. Colon and liver tissues were excised, rinsed with saline, and processed for further analysis. Distal colon tissues were immediately fixed in 4% paraformaldehyde and stored at 4 °C for subsequent paraffin embedding and sectioning. Portions of the colon and liver tissues were homogenized in nine volumes of saline and stored at 4 °C, with the remainder stored at −80 °C. All animal procedures were conducted in accordance with the ethical guidelines of the Ethical Review Committee for Laboratory Animal Welfare and Animal Experimentation of China Agricultural University (Permit No. Aw12402202-5-1; approval date: 10 January 2022).

### 2.3. Quantitative Analysis of Inflammatory Cytokines and Antioxidant Indices in Colon, Liver, and Serum

Colon and liver tissue homogenates were centrifuged at 12,000 rpm for 15 min at 4 °C. Protein concentrations were quantified using a BCA (bicinchoninic acid) Protein Assay Kit (Shanghai, China) according to the manufacturer’s instructions. Inflammatory cytokines (IL-6, IL-10, TNF-α) in the colon, liver, and serum were measured using ELISA kits, while oxidative stress markers (SOD, MDA, GSH, CAT, GPX, MPO, T-AOC) were assessed in the colon, liver, and serum using respective assay kits.

### 2.4. Histological Analysis

Colonic tissues fixed in 4% paraformaldehyde were processed into paraffin sections, deparaffinized, washed, and stained with hematoxylin and eosin (H&E). Inflammatory infiltrates were observed under a microscope and imaged using a Leica DM2000 imaging system.

### 2.5. Evaluation of the Pyroptosis Pathway

The levels of caspase-1, GSDMD, and IL-1β in colon and liver tissues were detected and quantified using ELISA kits.

### 2.6. Measurement of Liver Biomarkers

Serum levels of ALT, ALP, AST, and GGT were determined using ELISA kits.

### 2.7. Total RNA Extraction and RT-qPCR

Total RNA isolation from colonic and hepatic tissues was conducted utilizing the Nucleic Acid Extraction Kit (Beijing-based Tiangen Biotech Co., Ltd., China). Complementary DNA (cDNA) was generated via the FastKing cDNA First Strand reverse transcription kit (Tiangen Biotech, Beijing, China). Quantitative PCR amplification reactions were performed with SuperReal PreMix Plus reagents (Tiangen Biotech, China) on a CFX96 thermal cycler (Bio-Rad, Hercules, CA, USA), employing sequence-specific primers (Table 1). Target gene expression levels were calibrated against the reference gene GAPDH, with relative quantification achieved through the 2^−ΔΔCt^ algorithm [27].

### 2.8. Protein Immunoblotting of Mouse Colon and Liver Tissues

Colonic and hepatic tissue samples underwent mechanical disruption and solubilization in RIPA lysis buffer supplemented with protease/phosphatase inhibitors (PMSF, sodium orthovanadate) [28]. Following centrifugation, protein concentrations in the supernatants were subsequently quantified via BCA assay. Aliquots were subjected to electrophoretic separation and membrane transfer, after which membranes were incubated in a blocking solution of 5% non-fat dried milk. Primary antibodies were immunoblotted under overnight refrigeration at 4 °C, followed by subsequent probing with species-matched secondary antibodies for 60 min at ambient temperature. Protein bands were visualized via enhanced chemiluminescence (ECL) detection reagents (BioVision, San Francisco, CA, USA), with semi-quantitative analysis of band intensities performed using a Bio-Rad Gel Doc 2000 imaging platform (Hercules, CA, USA).

### 2.9. 16S rRNA Gene Sequencing

Frozen fecal samples were analyzed for gut microbial community structure using 16S rRNA sequencing. Total DNA was extracted, and the V3-V4 hypervariable regions of the 16S rRNA gene were amplified using universal primers 338F and 806R. Purified amplicons were subjected to paired-end sequencing on the Illumina MiSeq PE300 platform (Illumina, San Diego, CA, USA) following standard protocols from Shanghai Meiji Bio-pharmaceutical Technology Co., Ltd. (Shanghai, China). Operational taxonomic units (OTUs) were clustered with 97% similarity using UPARSE version 7.1 [29]. Representative sequences were classified against the 16S rRNA database using the RDP classifier version 2.231 [30] with a confidence threshold of 0.7%.

### 2.10. Correlation Analysis

Spearman correlation analysis was performed to assess the relationships between gut microbiota composition and LPS levels, as well as other biomarkers.

### 2.11. Statistical Analyses

Experimental results were expressed as mean values ± standard deviation (SD) and processed with GraphPad Prism (v8.0; GraphPad Software Inc., San Diego, CA, USA). Statistical comparisons employed one-way analysis of variance (ANOVA) supplemented with post hoc analyses using the Least Significant Difference (LSD) method. Statistical significance thresholds were set at *p* < 0.05.

## 3. Results and Discussion

### 3.1. Effects of HT on Disease Symptoms and Histopathological Features in Mice

**Figure 1:**
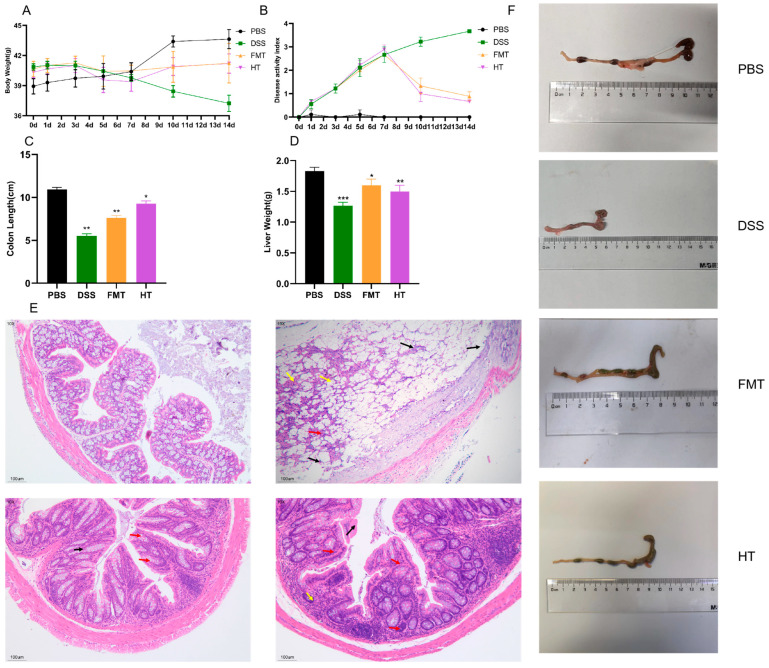
Effect of HT on symptoms of colitis in mice. (**A**) Body weight changes; (**B**) disease activity index (DAI) score; (**C**) colon length; (**D**) liver weight; (**E**) H&E stained sections of the colon; (**F**) colonic morphology. *** *p* < 0.001 vs. PBS, ** *p* < 0.01 vs. PBS, * *p* < 0.05 vs. PBS (*n* = 3).

In this investigation, a mouse model of colitis was established using a 3% solution of DSS to assess the potential therapeutic effects of HT on the condition [31]. The Disease Activity Index (DAI) scores and colon length served as primary metrics for evaluating HT’s impact on colitis in the mice. The DAI scores offer a thorough evaluation of colitis severity [32], taking into account variations in body weight, stool consistency, and the presence of blood in feces, with elevated scores reflecting more severe colitis. As depicted in Figure 1A, after seven days of DSS treatment, all treatment groups exhibited a reduction in body weight compared to the PBS group. However, the body weight in the FMT and HT groups showed a gradual recovery following an additional seven days of treatment. Figure 1B demonstrates that the DAI scores were highest in the DSS group, while the scores for the HT and FMT groups neared those of the PBS group post-treatment.

Moreover, Figure 1C,D,F reveal that the colon length was significantly reduced in the DSS group compared to the PBS group. In contrast, both the HT and FMT groups demonstrated a restoration of colon length following seven days of treatment. Additionally, DSS administration led to a decrease in liver mass in the mice, an effect that was alleviated in the HT and FMT groups.

Histological analysis of the mouse colon was conducted using H&E staining to evaluate tissue damage. Inflammatory cell infiltration, predominantly characterized by leukocyte infiltration, is a hallmark of most forms of colitis [33]. This infiltration can lead to several pathological changes, including thickening of the gut wall, compression of the gut lumen, formation of inflammatory pseudopolyps, and alterations in the muscularis layer. As illustrated in Figure 1E, colons from the PBS group exhibited normal morphology, characterized by an intact mucosal layer, numerous crypts, abundant goblet cells, a thin muscular layer, and no significant inflammatory cell infiltration. In contrast, mice treated with DSS displayed pronounced histopathological changes. These changes included significant epithelial damage, a reduction in goblet cells (indicated by red arrows), distorted crypt architecture, extensive inflammatory cell infiltration (indicated by black arrows), hyperchromatic nuclei, cellular suspension, crumpling, and apoptotic cell death (indicated by yellow arrows). However, treatment with FMT and HT led to the restoration of normal colonic tissue structure. This restoration was evidenced by an increased number of goblet cells, absence of edema, and reduced inflammatory cell infiltration in both groups, thereby confirming the efficacy of HT in ameliorating DSS-induced colonic injury.

### 3.2. Effects of HT on Inflammatory Factors and Antioxidant Indices in Mice

**Figure 2:**
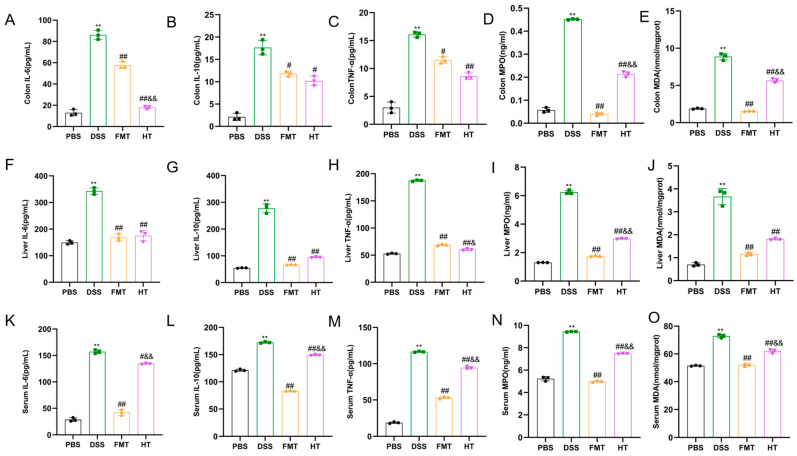
Hydroxytyrosol alleviates inflammatory response and oxidative stress in colitis mice. (**A**) Colon IL-6; (**B**) colon IL-10; (**C**) TNF-α; (**D**) liver IL-6; (**E**) liver IL-10; (**F**) liver TNF-α; (**G**) serum IL-6; (**H**) serum IL-10; (**I**) serum TNF-α; (**J**) colon MPO; (**K**) colon MDA; (**L**) liver MPO; (**M**) liver MDA; (**N**) serum MPO; (**O**) serum MDA. ** *p* < 0.01 vs. PBS ^##^ *p* < 0.01 vs. DSS ^#^ *p* < 0.05 vs. DSS ^&&^ *p* < 0.01 vs. FMT ^&^ *p* < 0.05 vs. FMT (*n* = 3).

In our study, as illustrated in Figure 2A–C,F–H,K–M, we investigated the changes in these inflammatory factors within mouse tissues. The results indicated that the levels of IL-6, IL-10, and TNF-α in the colon, serum, and liver were significantly elevated in the DSS group compared to the PBS group (*p* < 0.01). After seven days of treatment, the HT and FMT groups exhibited a decreasing trend in these inflammatory markers. Notably, the levels of IL-6, IL-10, and TNF-α in the liver and serum of mice in the HT and FMT groups were significantly lower than those in the DSS group (*p* < 0.01). Similarly, a decreasing trend was observed in the colon, with IL-6, IL-10, and TNF-α levels significantly reduced compared to the DSS group (*p* < 0.01, *p* < 0.05). Furthermore, the reduction in IL-6 and IL-10 levels in the colon and liver was more pronounced in the HT group compared to the FMT group. These results suggest that HT may alleviate inflammation by inhibiting the release of inflammatory factors.

**Figure 3:**
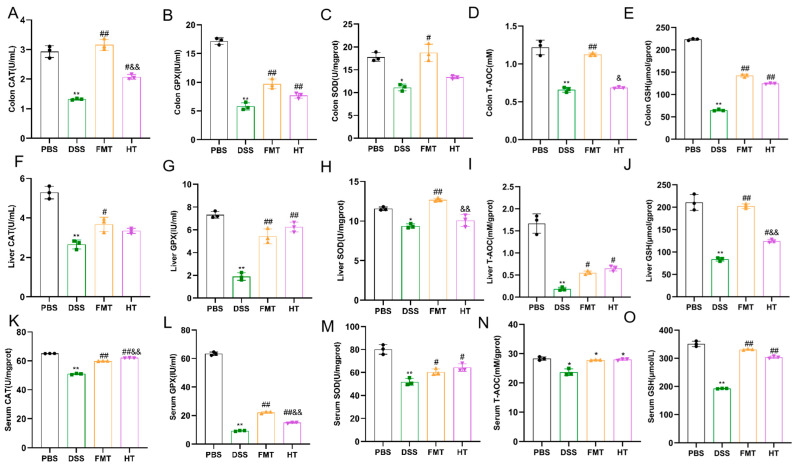
HT alleviates oxidative stress in colitis mice. (**A**) colon CAT; (**B**) colon GPX; (**C**) colon SOD; (**D**) colon T-AOC; (**E**) colon GSH; (**F**) liver CAT; (**G**) liver GPX; (**H**) liver SOD; (**I**) liver T-AOC; (**J**) liver GSH; (**K**) serum CAT; (**L**) serum GPX; (**M**) serum SOD; (**N**) serum T-AOC; (**O**) serum GSH. ** *p* < 0.01 vs. PBS, * *p* < 0.05 vs. PBS ^##^ *p* < 0.01 vs. DSS, ^#^ *p* < 0.05 vs. DSS ^&&^ *p* < 0.01 vs. FMT ^&^ *p* < 0.05 vs. FMT (*n* = 3).

In our study, we evaluated the effectiveness of HT by measuring oxidative stress markers, including CAT, GPX, MPO, SOD, MDA, GSH, and the antioxidant indicator T-AOC. As shown in Figure 2 and Figure 3, levels of MDA and MPO in the colon, liver, and serum were significantly higher in the DSS group compared to the PBS group (*p* < 0.01). In contrast, both the FMT and HT groups exhibited significantly lower levels of these markers compared to the DSS group (*p* < 0.01). Furthermore, the concentrations of CAT, GPX, SOD, GSH, and T-AOC in the colon, liver, and serum were markedly diminished in the DSS group relative to the PBS group (*p* < 0.01). Notably, T-AOC levels in the colon, liver, and serum were significantly reduced (*p* < 0.01, *p* < 0.05) in the DSS group compared to the PBS group. However, in the FMT group, T-AOC levels in both the colon and liver, as well as CAT levels in the HT group, were significantly elevated compared to the DSS group (*p* < 0.01, *p* < 0.05). These results indicate a substantial increase in oxidative stress in mice with colitis, characterized by elevated lipid peroxidation products such as MDA and MPO, which compromised the body’s antioxidant defenses and resulted in cellular damage.

### 3.3. Effects of HT on Colonic and Hepatic Pyroptosis in Mice

**Figure 4:**
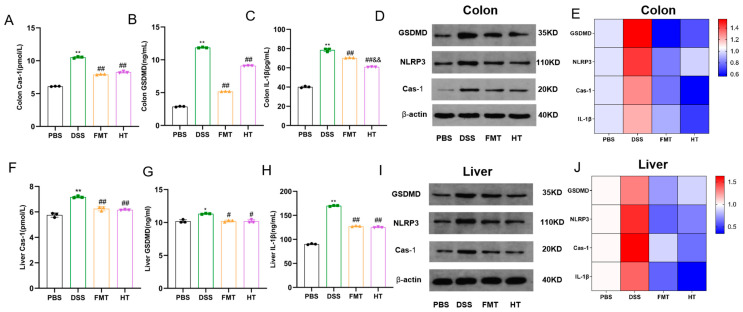
Hydroxytyrosol alleviates pyroptosis in colitis mice. (**A**) Colon Cas-1; (**B**) colon GSDMD; (**C**) colon IL-1; (**D**) Western blot analysis of the expression of NLRP3, Cas-1, and GSDMD in colon tissues; (**E**) expression of different genes in colon tissues; (**F**) liver Cas-1; (**G**) liver GSDMD; (**H**) liver IL-1β; (**I**) Western blot analysis of the expression of NLRP3, Cas-1, and GSDMD in liver tissues; (**J**) expression of different genes in liver tissues. ** *p* < 0.01 vs. PBS, * *p* < 0.05 vs. PBS, ^##^ *p* < 0.01 vs. DSS, ^#^ *p* < 0.05 vs. DSS, ^&&^ *p* < 0.01 vs. FMT, (*n* = 3).

This study investigates the impact of DSS-induced colitis on the expression levels of Cas-1, GSDMD, and IL-1β in mice, alongside evaluating the potential therapeutic effects of HT and FMT. Our findings indicate that the levels of Cas-1, GSDMD, and IL-1β in the colons of the DSS group were significantly elevated compared to the PBS group, as illustrated in Figure 4A–C (*p* < 0.01). These results were corroborated by Western blot analyses, which revealed similar trends in the expression of GSDMD, NLRP3, and Cas-1, as well as in the mRNA levels of the associated genes (Figure 4D,E). Furthermore, the liver tissues of the DSS group demonstrated significantly higher levels of Cas-1, IL-1β, and GSDMD compared to other groups (*p* < 0.01, *p* < 0.05), with hepatic NLRP3 levels reflecting those observed in the colon (Figure 4F–H). The quantitative analyses presented in Figure 4I,J further supported these patterns of change.

### 3.4. Effects of HT on Gut Inflammatory Markers, Inflammatory Gene Expression, and Gut Barrier and Hepatic Injury in Mice

**Figure 5:**
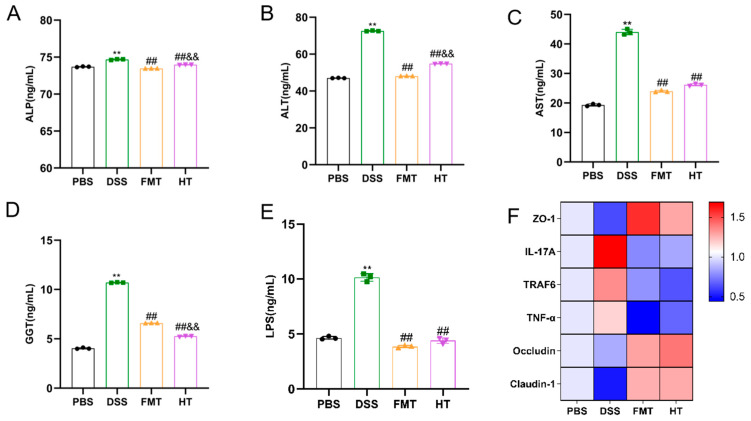
Effect of hydroxytyrosol on the expression of LPS, liver biomarkers, and gut barrier proteins in colitis mice. (**A**) ALP; (**B**) ALT; (**C**) AST; (**D**) GGT; (**E**) LPS; (**F**) Expression of gut barrier-related genes. ** *p* < 0.01 vs. PBS, ^##^ *p* < 0.01 vs. DSS, ^&&^ *p* < 0.01 vs. FMT, (*n* = 3).

In this study, we utilized ELISA kits to assess serum levels of ALT, AST, ALP, and GGT, which are widely recognized as diagnostic markers for hepatocellular injury. As illustrated in Figure 5A–D, the DSS group exhibited significantly elevated levels of ALP, ALT, AST, and GGT, suggesting substantial liver and kidney damage. In contrast, all treatment groups demonstrated significantly lower levels of these markers (*p* < 0.01) compared to the DSS group. This reduction in biochemical markers following intervention with HT highlights its protective role in maintaining liver and kidney function.

In our investigation, we evaluated the concentrations of the inflammatory marker LPS and observed that the DSS group displayed significantly elevated levels of LPS in comparison to the other groups (*p* < 0.01, Figure 5E). Moreover, we examined the expression of genes associated with inflammation and the integrity of the gut barrier. The relative mRNA levels of inflammatory markers IL-17A, TNF-α, and TRAF6 were significantly increased in the DSS group compared to the PBS group (*p* < 0.05, Figure 5F). In contrast, the HT group showed a notable decrease in the expression of these inflammatory genes when compared to the DSS group (*p* < 0.01). Furthermore, HT treatment resulted in the upregulation of genes responsible for tight junction proteins, including Occludin, ZO-1, and Claudin-1, which had been suppressed due to DSS exposure.

### 3.5. Effects of HT on the TLR4/NF-κB Signaling Pathway in DSS-Induced Colitis in Mice, and iNOS, COX-2, and TGF-β1 Expression Levels

**Figure 6:**
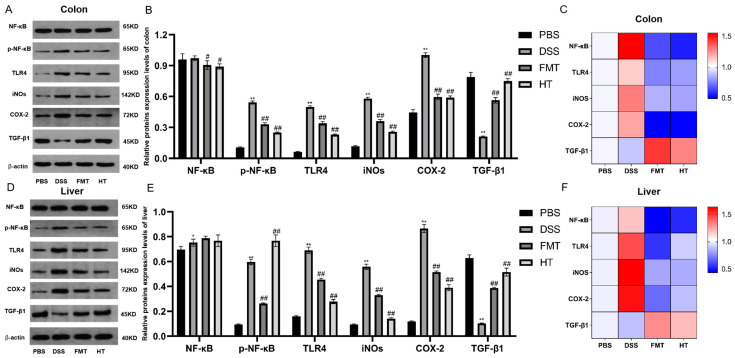
Effect of hydroxytyrosol on TLR4/NF-κB signaling pathway and iNOS, COX-2, and TGF-β1 in colon and liver tissues of colitis mice. (**A**) Western blot analysis of the expression of NF-κB, p-NF-κB, TLR4, iNOS, COX-2, TGF-β1 in colon tissues; (**B**) expression of genes in colon tissues; (**C**) relative expression of proteins in colon tissues; (**D**) Western blot analysis of the expression of NF-κB, p-NF-κB, TLR4, iNOS, COX-2, TGF-β1 in liver tissues; (**E**) expression of genes in liver tissues; (**F**) relative expression of proteins in in liver tissues. ** *p* < 0.01 vs. PBS, * *p* < 0.05 vs. PBS, ^##^ *p* < 0.01 vs. DSS, ^#^ *p* < 0.05 vs. DSS (*n* = 3).

Figure 6 illustrates the comparative analysis of protein levels among different groups. In the DSS group, there was a significant increase in the levels of TLR4 protein, iNOS, COX-2 protein, and phosphorylated NF-κB in both the colon and liver compared to the PBS group (*p* < 0.05). Conversely, the level of TGF-β1 protein was significantly lower in the DSS group. In contrast, the FMT and HT groups demonstrated a significant reduction in the levels of TLR4, iNOS, COX-2, and phosphorylated NF-κB proteins compared to the DSS group (*p* < 0.05). Additionally, the level of TGF-β1 protein was significantly elevated in these groups.

### 3.6. Effects of FCD on the Gut Microbiota of Mice

In this research, we examined the diversity of fecal microbiota in experimental mice through advanced molecular biology methods. We extracted total DNA from 12 fecal samples and amplified the V3-V4 region of the 16S rRNA gene, which was subsequently sequenced using the Illumina MiSeq platform (Shanghai Meiji Biomedical Technology Co., Ltd., Shanghai, China). After implementing stringent quality control protocols, we acquired 788,664 optimized sequences with an average length of 420 base pairs. This approach facilitated the identification of 1885 OTUs, ensuring thorough sample coverage and sufficient species richness.

**Figure 7:**
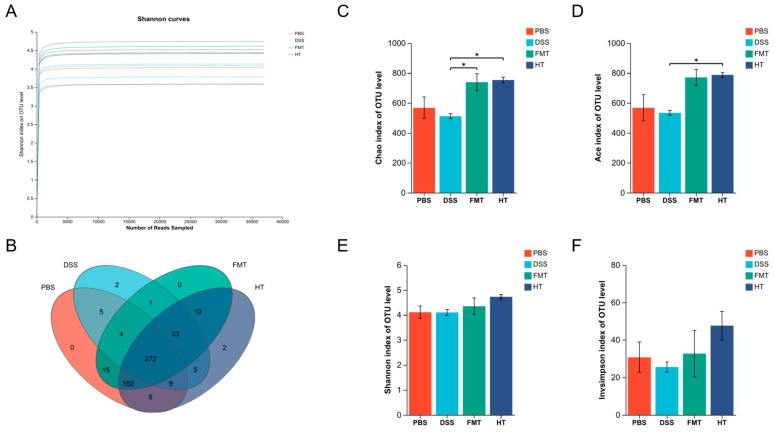
Effect of hydroxytyrosol on gut microbiota in colitis mice. (**A**) Rarefaction curves of OTUs clustered at 97% sequence similarity across different groups; (**B**) OTU Venn diagram; (**C**) Chao index; (**D**) Ace index; (**E**) Shannon index; (**F**) insimpson index. * *p* < 0.05 (*n* = 3).

The rarefaction curves for the species eventually plateaued, suggesting that the sequencing data obtained for these samples was sufficient (Figure 7A). An analysis of OTUs revealed that the PBS, DSS, FMT, and HT groups had 413, 331, 437, and 439 OTUs, respectively. Notably, the DSS group exhibited fewer OTUs compared to the other groups, indicating a decline in microbial diversity as a result of DSS treatment (Figure 7B). Both FMT and HT treatments were effective in reversing this decline. The Ace index, which measures community richness, showed a significant difference between the DSS and HT groups (*p* < 0.05). Similarly, the Chao index, another measure of community richness, demonstrated significant differences between the DSS and FMT groups and the HT group (Figure 7C,D, *p* < 0.05). In contrast, the Shannon and inverse Simpson alpha diversity indices, which assess community diversity, did not reveal significant differences between the groups (Figure 7E,F, *p* > 0.05). These findings imply that while HT did not significantly alter the diversity of the gut microbiota, both FMT and HT increased the abundance of gut microorganisms. Overall, the results suggest that HT alleviated the DSS-induced reduction in gut microbial diversity, potentially serving a protective role in maintaining gut microbiota health.

**Figure 8:**
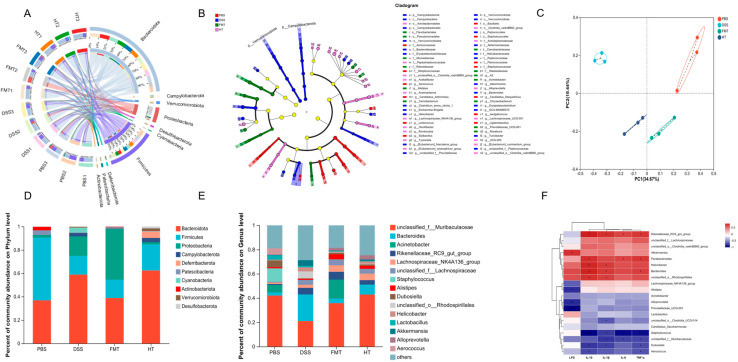
Effect of HT on gut microbiota in colitis mice. (**A**) Circos analysis; (**B**) LEfSe analysis; (**C**) PCoA analysis; (**D**) the relative abundance plot in the bacterial phylum level of the gut microbiota; (**E**) the relative abundance plot in the bacterial genus level of the gut microbiota; (**F**) Spearman correlation analysis. *** *p* < 0.001 vs PBS, ** *p* < 0.01 vs. PBS * *p* < 0.05 (*n* = 3).

To assess the effect of HT on gut microbial composition, we conducted Circos analysis at the phylum level (Figure 8A). The results indicated that Bacteroidota, Firmicutes, and Proteobacteria were the dominant phyla present in all four groups, although their relative abundances varied between the groups. Such variations in gut microbial diversity often result in changes in microbial abundance. To further explore these differences, we employed linear discriminant analysis of effect size (LEfSe) to evaluate the relative abundance of significant taxa between the groups (Figure 8B). Our findings indicated that the PBS group was characterized by a higher abundance of *Staphylococcaceae* and *Staphylococcales*, while the DSS group showed a predominance of *Bacteroidaceae* and *Bacteroides*. In contrast, the FMT group was enriched with *Moraxellaceae* and *Pseudomonadales*, and the HT group exhibited a dominance of *Lachnospiraceae_NK4A136_group* and *Alloprevotella*.

Principal coordinate analysis (PCoA) demonstrated notable variations in bacterial community abundance among the samples (Figure 8C). The DSS model group and the FMT group exhibited greater dispersion from the PBS group, indicating substantial alterations in gut microbial structure compared to the blank group. In contrast, the HT group showed a closer proximity to the PBS group, suggesting that HT may facilitate the restoration of gut microbial abundance and structure.

At the phylum level, ten phyla were identified, with Bacteroidota, Firmicutes, Proteobacteria, Campylobacterota, and Verrucomicrobiota being the most dominant (Figure 8D,E). In the DSS group, Bacteroidota abundance was higher compared to the PBS group, but it decreased following FMT and HT interventions. Conversely, Firmicutes abundance was lower in the DSS group than in the other groups, suggesting a DSS-induced decrease that was reversed by HT and FMT treatments. At the genus level, 34 microbial communities with relative abundance greater than 1% were identified. Among these, the dominant genera included *unclassified_f__Muribaculaceae*, *Bacteroides*, *Acinetobacter*, *Rikenellaceae_RC9_gut_group*, and *Lachnospiraceae_NK4A136_group*. The relative abundance of these genera varied across treatment groups.

To explore the connection between gut microbiota and inflammatory factors, we conducted Spearman correlation analyses examining the levels of inflammatory markers in the colon, the presence of colitis, gut microbiota composition, and gut metabolites across four experimental groups (Figure 8F). The analysis revealed several significant correlations at the genus level. Notably, the relative abundance of *Rikenellaceae_RC9_gut_group* showed significant positive correlations with IL-1β, IL-6, and TNF-α, while *unclassified_f__Muribaculaceae* exhibited significant negative correlations with IL-1β and TNF-α. The relative abundance of *Staphylococcus* demonstrated significant negative correlations with IL-1β, IL-6, and TNF-α. *Helicobacter* and *unclassified_o__Rhodospirillales* were significantly positively correlated with IL-1β, whereas *unclassified_o__Clostridia_UCG-014* showed a significant negative correlation with IL-1β. *Bacteroides* and *Dubosiella* displayed significant positive and negative correlations with IL-1β and TNF-α, respectively. *Parabacteroides* exhibited significant positive correlations with IL-6 and TNF-α, while *Aerococcus* was significantly negatively correlated with TNF-α. Additionally, *Akkermansia* showed a significant positive correlation with LPS, whereas *Alistipes*, *Alloprevotella*, and *Prevotellaceae_UCG-001* demonstrated significant negative correlations with LPS. These findings highlight the complex interactions between specific gut microbiota and inflammatory markers.

## 4. Discussion

This study investigated the multifaceted protective effects of HT against DSS-induced colitis, demonstrating its therapeutic efficacy through suppressing inflammatory responses, modulating oxidative stress, restoring intestinal barrier integrity, and rebalancing gut microbiota homeostasis. These findings provide a novel therapeutic strategy utilizing natural products for the treatment of IBD.

When evaluating the therapeutic effects of HT on body weight and colonic function in DSS-treated mice, our study revealed that HT administration alleviated DSS-induced weight loss, colon shortening, and decline in liver weight. Histological analysis further demonstrated that HT intervention increased the number of goblet cells in colonic tissues while reducing edema and inflammatory cell infiltration, indicating its capacity to repair intestinal mucosal damage.

According to fundamental studies, the primary pathological mechanism of colitis [34] involves persistent oxidative stress and inflammatory responses at lesion sites, leading to gastrointestinal endothelial cell damage. When excessive free radicals overwhelm the antioxidant defense system, the physiological balance is disrupted, resulting in oxidative stress. This state disrupts the equilibrium between pro-oxidants and antioxidants, causing cellular injury. Elevated oxidative stress levels in tissues further trigger cascading inflammatory reactions characterized by overexpression of inflammatory cytokines. During inflammation, TNF-α, IL-6, and IL-10 are three pivotal cytokines [35]. Furthermore, the body’s evolved antioxidant defense system plays a critical role in countering oxidative stress through enzymatic antioxidants such as SOD, CAT, and GPX. SOD [36] is particularly vital as it scavenges superoxide radicals, thereby preventing the formation of more harmful peroxynitrite and maintaining physiologically relevant nitric oxide levels. In addition to enzymatic antioxidants, the non-enzymatic antioxidant glutathione (GSH) serves as the primary endogenous antioxidant, participating in diverse biological processes and providing protection against oxidative damage [37]. Our study revealed that colitis mice exhibited significantly elevated oxidative stress levels. However, HT intervention therapeutically downregulated inflammatory cytokines (TNF-α, IL-6, and IL-10) and oxidative stress markers. Concurrently, HT augmented the activities of antioxidant enzymes (SOD, CAT) and elevated GSH levels, effectively restoring the pro-oxidant/antioxidant balance. These findings demonstrate that HT regulates oxidative stress biomarkers, enhances endogenous antioxidant capacity, and contributes to maintaining systemic homeostasis.

At the regulatory level of pyroptosis, the DSS group exhibited elevated levels of Cas-1 and GSDMD in the colon and liver, while HT treatment effectively suppressed NLRP3 inflammasome activation and reduced levels of Cas-1, GSDMD, and the downstream effector IL-1β. Pyroptosis is a form of programmed cell death characterized by continuous cell swelling until membrane rupture, leading to the release of cellular contents and subsequent activation of intense inflammatory responses. Cas-1 and GSDMD are key proteins in pyroptosis. Cas-1, a core component of the inflammasome-dependent pyroptotic pathway, can be activated by the canonical NLRP3 inflammasome [38]. Upon stimulation from microbial infections, oxidative stress, or other factors, Cas-1 senses danger through the inflammasome, recruits and activates caspase-1, which in turn cleaves and activates inflammatory cytokines such as IL-18 and IL-1β. IL-1β, a critical mediator of antimicrobial immunity and autoimmune inflammation, acts as a potent pro-inflammatory factor, promoting the proliferation and release of inflammatory cells, which is essential for host defense against pathogens [39]. The inhibitory effect of HT on these key nodes may alleviate inflammatory damage by blocking the pyroptosis pathway. HT also reduced serum levels of liver function indicators such as ALP, ALT, AST, and GGT [40], indicating its hepatoprotective effects and further expanding its multi-organ protective potential.

Lipopolysaccharide [41], a highly immunogenic particle present in the outer membrane of Gram-negative bacteria, can trigger inflammatory cascades. The DSS group showed significantly elevated LPS levels in mice, suggesting the induction of systemic inflammation. IL-17 [42], a key initiator of T cell-mediated inflammatory responses, amplifies inflammatory reactions by releasing inflammatory cytokines. Th17 cells secrete pro-inflammatory factors such as IL-17A, IL-17F, IL-6, and TNF-α. TRAF serves as a critical mediator in IL-17 cytokine signaling, facilitating inflammatory and apoptotic pathways that broadly participate in immune and inflammatory responses. Tight junction (TJ) proteins, a major form of intercellular connections, are composed of structural proteins (e.g., Claudins, Occludin, JAM, ZOs) and various junctional molecules, forming the core structure of the mucosal mechanical barrier. Loss of barrier integrity may contribute to colitis pathogenesis. Study results demonstrate that HT upregulates tight junction proteins and downregulates inflammation-related gene expression, partially restoring intestinal barrier function and mitigating inflammatory responses.

TGF-β1 is an anti-inflammatory cytokine known for regulating cell growth and differentiation, with multiple functions including suppressing lymphocyte proliferation, macrophage activation, and overall inflammatory responses [36,42]. The TLR4/NF-κB signaling pathway is intricately involved in cytokine production, where NF-κB serves as a pivotal component in this process [43]. Under normal conditions, effector proteins of the TLR4/NF-κB pathway, such as p65, p52, and IκB, form a stable complex in the cytoplasm. However, exogenous signals, such as inflammation, can activate the TLR4 receptor, leading to phosphorylation of NF-κB. The phosphorylated NF-κB subsequently translocates to the nucleus, where it regulates the expression of target genes, stimulating the production of pro-inflammatory cytokines like IL-6 and TNF-α, as well as other inflammatory mediators such as iNOS and COX-2, thereby promoting inflammation. Our experimental results demonstrated that HT treatment significantly reduced the expression levels of TLR4, NF-κB (p65), phosphorylated NF-κB (p-p65), iNOS, and COX-2 in mouse colon and liver tissues. Concurrently, HT treatment increased TGF-β1 expression levels. These findings suggest that HT may alleviate inflammatory responses in colitis by suppressing the NF-κB signaling pathway, thereby reducing the release of inflammatory factors and mitigating inflammation.

It is currently known that the gut microbiota plays a crucial role in regulating immune responses, aiding digestion, promoting neural signaling, facilitating angiogenesis, and defending against pathogens. Dysbiosis, or imbalance in the gut microbiota, can lead to various diseases, such as cancer and cardiovascular disorders. Considering this, we performed 16S rRNA sequencing to further explore the restorative effects of HT on the gut microbiota in DSS-induced colitis mice [44]. Our findings revealed that, compared to the DSS group, HT intervention decreased the relative abundance of Bacteroidota and increased the relative abundance of Firmicutes. Previous studies have shown that *Bacteroidetes* is associated with the expression of pro-inflammatory factors [45], while an increased abundance of Firmicutes often enhances the production of the anti-inflammatory cytokine IL-10 [46]. Our results align with these reports, suggesting that the observed reduction in pro-inflammatory factors and elevation in anti-inflammatory factors in the HT-treated group may be linked to the altered abundances of Bacteroidota and Firmicutes. Studies indicate that *Alistipes* [47] is typically present in the gastrointestinal tract of healthy individuals. In our study, the DSS group exhibited reduced *Alistipes* abundance and increased Bacteroides levels, a genus linked to pro-inflammatory factor secretion. Following FMT and HT treatment, *Alistipes* abundance increased while *Bacteroides* decreased. Furthermore, *Alistipes* showed a significant negative correlation with LPS, whereas *Bacteroides* correlated positively with IL-1β and TNF-α. Notably, LPS [48] can induce the secretion of inflammatory cytokines. Additionally, the study found that *unclassified_f__Muribaculaceae* was less abundant in the DSS group. This taxon is negatively associated with the inflammatory state of colitis [49], and its abundance is closely tied to propionic acid, which suppresses CD8+ T cell activation to tolerate immune stimulation. These findings suggest that HT may alleviate colitis by modulating the relative abundances of *Alistipes*, *Bacteroides*, and *unclassified_f__Muribaculaceae*. Together, these results provide insights into the potential mechanisms by which HT influences the composition and diversity of the gut microbiota in DSS-induced colitis.

## 5. Conclusions

This study highlights the potential of HT in preventing DSS-induced colitis and investigates the underlying mechanisms involved. HT effectively reduces colitis symptoms, improves colonic histopathology, enhances antioxidant and anti-inflammatory responses, and protects the colon from damage. Oral administration of HT mitigates DSS-induced colitis by regulating the NLRP3-Cas1-GSDMD-IL-1β inflammatory signaling pathway within the gut mucosal barrier and by inhibiting the NF-κB inflammatory pathway. Additionally, HT promotes the expression of TJ proteins, including claudin-1, occludin, and ZO-1, which contribute to the regeneration of the gut barrier and help maintain intestinal homeostasis. Moreover, there is a recognized connection between colitis and liver injury, particularly in colitis patients. The liver, which plays a crucial role in filtering and processing inflammatory waste products, can become overwhelmed by the increased inflammatory load caused by colitis, potentially leading to liver damage. HT also restores gut microbial diversity and enhances microbial abundance, further attenuating colitis. The findings of this study provide scientific evidence supporting the beneficial effects of HT on gut health.

In the future, HT could be developed as a functional food or a gut microbiota modulator combined with probiotics, offering novel therapeutic strategies for colitis drug development. Personalized intervention strategies integrating gut microbiota profiling may enhance clinical efficacy. Further research can validate HT’s long-term safety in large animal models to advance the development of natural product-based therapies for colitis.

## Figures and Tables

**Table 1:** The primer sequences in RT-qPCR.

Gene	Forward (5′-3′)	Reverse (3′-5′)
TNF-α	ATGAGCACAGAAAGCATGA	AGTAGACAGAAGAGCGTGGT
IL-1β	TCCAGGATGAGGACATGAGCAC	GAACGTCACACACCAGCAGGTTA
COX-2	CCCCATTAGCAGCCAGTT	CATTCCCCACGGTTTTGA
iNOS	GTTCTCAGCCCAACAATACAA	GTGGACGGGTCGATGTCACGA
IL-10	ATAACTGCACCCACTTCCCA	GGGCATCACTTCTACCAGGT
TGF-β1	CCTGCAAGACCATCGACATG	TGTTGTACAAAGCGAGCACC
NF-kB	GTGGTGCCTCACTGCTAACT	GGATGCACTTCAGCTTCTGT
IL-17A	GGAAAGGACGGACTGGTGTA	TGCCACTGGTCTGTAATCCA
TRAF6	GTATCCGCATTGAGAAGC	GCAGTGAACCATCCGTGT
Occludin	GAGGAGAGTGAAGAGTACATGGGCTG	GTCTGTCATAATCTCCCACCATCCT
ZO-1	TCATCCCAAATAAGAACAGAGC	GAAGAACAACCCTTTCATAAGC
Claudin-I	TCCTTGCTGAATCTGAACA	AGCCATCCACATCTTCTG
Caspase-1	TGCCCAGAGCACAAGACTTC	TCCTTGTTTCTCTCCACGGC
GSDMD	ATGGCATGGCTTACACCACC	ATGGCATGGCTTACACCACC
NLRP3	TCTGCACCCGGACTGTAAAC	CACCCAACTGTAGGCTCTGC
β-actin	AAGTCCCTCACCCTCCCAAAAG	AGCAATGCTGTCACCTTCCC

## Data Availability

The original contributions presented in the study are included in the article, further inquiries can be directed to the corresponding author.

## Abbreviations

The following abbreviations are used in this manuscript:

HT: hydroxytyrosol; IBD: inflammatory bowel disease; UC: ulcerative colitis; TLR4: toll-like receptor 4; DSS: dextrose sodium sulfate; DPEC: paraformaldehyde; PBS: phosphate-buffered saline; ELISA: enzyme-linked immunosorbent assay; Cas-1: caspase-1; GSH: glutathione; MDA: malondialdehyde; SOD: superoxide dismutase; NO: nitric oxide; CAT: catalase; T-AOC: total antioxidant capacity; LPS: lipopolysaccharide; ALT: alanine aminotransferase; AST: aspartate aminotransferase; ALP: alkaline phosphatase; GGT: γ-glutamyltransferase; mRNA: messenger ribonucleic acid; GAPDH: glyceraldehyde-3-phosphate dehydrogenase (GAPDH); DAI: disease activity index; TJ: tight junction.
